# Cost-Effectiveness of Left Ventricular Assist Device for Transplant-Ineligible Patients

**DOI:** 10.1001/jamanetworkopen.2025.4483

**Published:** 2025-04-18

**Authors:** Elisabeth M. Schaffer, Rebecca Giok Sim Su, Junxing Chay, Eric A. Finkelstein

**Affiliations:** 1 Saw Swee Hock School of Public Health, National University of Singapore, Singapore; 2 Health Services and Systems Research, Duke-NUS Medical School, Singapore

## Abstract

**Importance:**

Singapore is considering subsidizing left ventricular assist devices (LVADs) for end-stage heart failure (ESHF) and uses cost-effectiveness evidence to inform the determination. Yet, no economic evaluation has thus far been conducted.

**Objective:**

To estimate the lifetime cost-effectiveness of LVAD compared with optimal medical management for transplant-ineligible patients.

**Design, Setting, and Participants:**

This economic evaluation used a Markov model to simulate survival, stroke incidence, other adverse events, and heart failure hospitalizations for a cohort of adult patients in Singapore with transplant-ineligible ESHF, most of whom were inotrope dependent. Latest LVAD mortality data from a randomized clinical trial were age-adjusted, and an indirect comparison of prior trial results was performed to estimate survival for inotrope-dependent and inotrope-independent patients. Costs were estimated (in 2023 Singapore dollars [SGD]) using cohort billing data from 2017 to 2022 and National Heart Centre Singapore LVAD charges. Statistical analysis was performed from December 2023 to July 2024.

**Exposure:**

HeartMate 3 LVAD (Abbott).

**Main Outcomes and Measures:**

Health care costs, quality-adjusted life-years (QALYs), and incremental cost-effectiveness ratio (ICER) evaluated against a threshold of SGD 114 000 (US $85 075) per QALY gained.

**Results:**

At model initiation, the cohort had a mean (SD) age of 64 (12) years, and 78% (range, 68%-86%) of patients were inotrope dependent. In the base case analysis, LVAD yielded an additional 3.45 QALYs at an incremental cost of SGD 404 678 (US $301 999), producing an ICER of SGD 117 370 (US $87 590) per QALY gained for the transplant-ineligible population. The ICER differed for inotrope-use subgroups at SGD 106 458 (US $79 446) per QALY gained for inotrope-dependent patients and SGD 174 798 (US $130 446) per QALY gained for inotrope-independent patients (with 59% and 19% probabilities, respectively, of attaining high value). The inotrope-dependent ICER was sensitive to model input changes and structural assumptions, whereas the inotrope-independent ICER consistently exceeded the high-value threshold in scenario analyses. In threshold analyses, a 44% reduction in the total implantation price or a 54% reduction in the all-cause mortality hazard were required for LVAD to be high value for inotrope-independent patients. Confidence that the inotrope-dependent ICER is high value increased to 75% and 85% with respective 20% and 33% reductions in total implantation price.

**Conclusions and Relevance:**

In this economic evaluation comparing LVAD with optimal medical management, LVAD was potentially high value for most transplant-ineligible patients who are inotrope dependent. Confidence in this result was improved with plausible price reductions, yet only extreme changes rendered LVAD high value for inotrope-independent patients.

## Key Points

**Question:**

Is left ventricular assist device (LVAD) cost-effective compared with optimal medical management for patients with transplant-ineligible end-stage heart failure?

**Findings:**

In this economic evaluation of a simulated cohort of adult patients in Singapore, LVAD's cost per quality-adjusted life-year gained was 106 458 in Singapore dollars (SGD; US $79 446) for inotrope-dependent patients and SGD 174 798 (US $130 446) for inotrope-independent patients, with 59% and 19% probabilities of attaining cost-effectiveness, respectively.

**Meaning:**

The findings suggest that LVAD is likely marginally cost-effective for inotrope-dependent patients, and confidence in this result would improve substantially with reductions in total implantation price.

## Teaser

This economic evaluation study estimates the health care costs, quality-adjusted life-years, and incremental cost-effectiveness ratio of a commonly used left ventricular assist device among Singaporean patients with end-stage heart failure.

## Introduction

Heart failure prevalence in Singapore is above the global mean at 4.5% and increasing due to higher prevalence of cardiovascular risk factors, rapid population aging, and recent drug therapy advances that have improved survival in early-stage disease.^1,2,3^ Yet, limited treatment options exist for an increasing number of patients with end-stage heart failure (ESHF). A severe shortage of donor hearts restricts heart transplantation to a select subset of patients, and many of these patients are transplant-ineligible due to advanced age or comorbidities.^4^ In 2012, durable left ventricular assist devices (LVADs) were introduced as another treatment option for transplant-ineligible patients. The Singaporean government is considering subsidizing LVADs yet requires evidence of clinical benefit and cost-effectiveness.^5^

Superior survival with LVAD compared with optimal medical management (MM) has been demonstrated in international randomized clinical trials. Compared with a 25% probability for MM, first-generation LVAD had a 1-year survival probability of 52% when used as destination therapy (DT; ie, treatment until death), which increased to 68% for second-generation LVAD.^6,7^ Survival with latest-generation devices is now measured in longer-term outcomes, with the latest trial reporting a 5-year survival of 52%.^8^ In addition to improved survival, quality-of-life gains and reduced adverse events (AEs) with the latest devices have established the clinical benefit of LVAD over MM.^9,10^

Whether LVAD delivers clinical benefit on par with health care resources expended is, however, unclear. No local cost-effectiveness analysis has been conducted, and international cost-effectiveness evidence is inconclusive. Although the first incremental cost-effectiveness ratio (ICER) for LVAD greatly exceeded conventional cost-effectiveness thresholds at US $802 700 per quality-adjusted life-year (QALY) gained (in 2002 US dollars, estimated in the US health care setting), much lower ICERs have since been estimated.^11^ ICERs for second-generation devices were less than 25% of the initial estimate, and the latest-generation devices have achieved even lower ICERs yet still above conventional thresholds.^12,13,14,15^

As health care costs in Singapore tend to be lower than those in other high-income countries, it is possible that LVAD is cost-effective in the city-state.^16^ HeartMate 3 (Abbott), hereafter called LVAD, is the most common LVAD used. In this study, we aimed to estimate the lifetime cost-effectiveness of LVAD compared with MM for transplant-ineligible patients using the health care system perspective.

## Methods

The National University of Singapore Institutional Review Board deemed this economic evaluation exempt from ethics review and informed consent because it was not human participant research. We followed the Consolidated Health Economic Evaluation Reporting Standards (CHEERS) reporting guideline.^17^

### Model Structure

We developed a Markov model to compare the costs and health outcomes associated with LVAD and MM for 15 years using Excel, version 2502 (Microsoft Corp). The study cohort comprised patients with ESHF—characterized by an ejection fraction less than 25% and New York Heart Association (NYHA) class III to IV symptoms that are refractory to guideline-directed medical therapy—and who are transplant-ineligible due to advanced age (≥60 years) or disqualifying comorbidities (eg, cancer, pulmonary disorders, active infection, autoimmune disorders, and substance use disorders).^18^

For both treatment groups (LVAD and MM), stroke is a severe AE that carries a long-term increased risk of mortality and morbidity. The model, therefore, included 2 alive states (alive with ESHF and alive after stroke with ESHF) and 1 dead state (eFigure 1 in Supplement 1). All patients entered the model in the alive with ESHF state, and monthly cycles captured transitions between health states and the incidence of short-term AEs. We modeled health state transitions separately for inotrope-dependent and inotrope-independent patients because survival and treatment costs differ for these subgroups.

### Survival

Although limited in sample size, outcome evidence from a Singapore study of transplant-ineligible patients implanted with LVAD suggests comparable survival to other high-income settings, according to KL Kerk, RN, BN, at the National Heart Centre Singapore (email, July 8, 2022). However, no randomized clinical trial has compared LVAD with MM in any country setting. In the absence of a head-to-head comparison, an indirect comparison using results from multiple trials that share 1 or more referent groups is recommended. Global trial evidence for the DT population includes recent 5-year survival outcomes for the latest-generation LVAD compared with the second-generation LVAD (HeartMate II; Abbott), which has been evaluated against an earlier-generation device (HeartMate XVE; Abbott) that, in turn, has been compared against MM.^6,7,8^ We used these trial results to estimate survival for transplant-ineligible patients in Singapore. Additionally, we used trial subgroup analyses and results from the ROADMAP (Risk Assessment and Comparative Effectiveness of Left Ventricular Assist Device and Medical Management in Ambulatory Heart Failure) trial to differentiate survival according to inotrope use (Table 1).^8,9,19^

**Table 1.** Model Inputs

	Base case input (range) [SE/SE]	Probabilistic sensitivity analysis distribution	Source
Population characteristics			
Age, mean (SD), y	64 (NV) [12]	Constant	Personal communication (email from KL Kerk, RN, BN, National Heart Centre Singapore, July 8, 2022)
Proportion inotrope dependent (SD)	0.78 (0.68-0.86) [0.08]	β	Rose et al,^6^ 2001; Slaughter et al,^7^ 2009; Goldstein et al,^10^ 2020
Survival			
LVAD			
Weibull scale parameter (SE)	0.03 (0.03-0.04) [0.01]	γ	Mehra et al,^8^ 2022
Weibull shape parameter (SE)	0.74 (0.62-0.86) [0.06]	β	Mehra et al,^8^ 2022
HR: inotrope independent vs MM inotrope independent (SD)	0.43 (0.43-0.77) [0.20]	Lognormal	Mehra et al,^8^ 2022; Starling et al,^9^ 2017; Stevenson et al,^19^ 2004
Probability of death after stroke in ESHF, mean (SD), mo	0.05 (0.02-0.07) [0.01]	β	Colombo et al,^20^ 2019
MM			
HR: full population vs LVAD (SD)	4.66 (3.75-4.66) [0.12]	Lognormal	Rose et al,^6^ 2001; Mehra et al,^8^ 2022; Goldstein et al,^10^ 2020
HR: inotrope dependent vs full population (SD)	1.36 (1.00-1.36) [0.06]	Lognormal	Rose et al,^6^ 2001; Stevenson et al,^19^ 2004
HR: inotrope independent vs full population (SD)	0.48 (0.40-1.00) [0.06]	Lognormal	Rose et al,^6^ 2001; Stevenson et al,^19^ 2004
Probability of death after stroke in ESHF, mean (SD), mo	0.08 (0.04-0.12) [0.02]	β	Appelros et al,^21^ 2003
AE probabilities, mean (SD), mo			
LVAD			
Stroke	0.007 (0.005-0.013) [0.001]	β	Goldstein et al,^10^ 2020
Major infection	0.068 (0.051-0.135) [0.014]	β	Goldstein et al,^10^ 2020
Bleeding	0.057 (0.043-0.113) [0.011]	β	Goldstein et al,^10^ 2020
Right HF	0.016 (0.008-0.024) [0.003]	β	Goldstein et al,^10^ 2020
Right HF, managed with RVAD	0.002 (0.001-0.004) [0.0005]	β	Goldstein et al,^10^ 2020
Pump thrombosis^a^	0.001 (0.000-0.001) [0.0002]	β	Goldstein et al,^10^ 2020
Worsening HF^b^	0.025 (0.012-0.037) [0.005]	β	Goldstein et al,^10^ 2020
MM: inotrope dependent			
Stroke	0.007 (0.004-0.011) [0.001]	β	Rose et al,^6^ 2001
Major infection	0.025 (0.012-0.037) [0.005]	β	Rose et al,^6^ 2001
Bleeding	0.005 (0.002-0.007) [0.001]	β	Rose et al,^6^ 2001
Right HF	0.002 (0.001-0.002) [0.0003]	β	Starling et al,^9^ 2017
Worsening HF^b^	0.173 (0.087-0.260) [0.035]	β	Rose et al,^6^ 2001
MM: inotrope independent			
Stroke	0.002 (0.001-0.004) [0.0005]	β	Starling et al,^9^ 2017
Major infection	0.025 (0.012-0.037) [0.005]	β	Rose et al,^6^ 2001
Bleeding	0.002 (0.001-0.002) [0.0003]	β	Starling et al,^9^ 2017
Right HF	0.002 (0.001-0.002) [0.0003]	β	Starling et al,^9^ 2017
Worsening HF^b^	0.091 (0.045-0.136) [0.018]	β	Starling et al,^9^ 2017
Health state utilities, mean (SE)			
LVAD: alive without stroke	0.767 (0.708-0.813) [0.153]	β	Goldstein et al,^10^ 2020; Göhler et al,^22^ 2009
LVAD: alive after stroke	0.290 (0.185-0.767) [0.058]	β	Rebchuk et al,^23^ 2020; Mehra et al,^24^ 2019
MM: inotrope dependent	0.532 (0.532-0.567) [0.106]	β	Göhler et al,^22^ 2009
MM: inotrope independent	0.603 (0.567-0.673) [0.121]	β	Göhler et al,^22^ 2009
MM: alive after stroke	0.487 (0.185-0.567) [0.097]	β	Rebchuk et al,^23^ 2020; Colombo et al,^20^ 2019
Utility decrement due to AE, %	10 (0-50) [0.020]	β	Assumption
Costs, mean (SD), 2023 SGD^c^			
LVAD	160 687 (80 005-165 709) [32 137]	γ	Personal communication (email from KL Kerk, RN, BN, National Heart Centre Singapore, November 18, 2022, and February 28, 2023); Malhotra et al,^25^ 2018
LVAD implantation procedure	186 021 (54 547-497 289) [37 204]	γ	Personal communication (email from KL Kerk, RN, BN, National Heart Centre Singapore, November 18, 2022, and February 28, 2023)
LVAD postoperative care	1045 (NV) [209]	γ	Personal communication (email from KL Kerk, RN, BN, National Heart Centre Singapore, November 18, 2022, and February 28, 2023)
Major infection	7407 (1449-23 080) [1481]	γ	Personal communication (email from KL Kerk, RN, BN, National Heart Centre Singapore, November 18, 2022, and February 28, 2023); Malhotra et al,^25^ 2018
Bleeding	6255 (3280-8495) [827]	γ	Malhotra et al,^25^ 2018
Right HF	7992 (NV) [1598]	γ	Personal communication (email from KL Kerk, RN, BN, National Heart Centre Singapore, November 18, 2022, and February 28, 2023)
RVAD (ie, temporary device)	7030 (NV) [1406]	γ	Personal communication (email from KL Kerk, RN, BN, National Heart Centre Singapore, November 18, 2022, and February 28, 2023)
Stroke	10 875 (3650-21 547) [1299]	γ	Personal communication (email from KL Kerk, RN, BN, National Heart Centre Singapore, November 18, 2022, and February 28, 2023)
Rehabilitation for stroke, cost per mo	740 (NV) [148]	γ	Personal communication (email from KL Kerk, RN, BN, National Heart Centre Singapore, November 18, 2022, and February 28, 2023)
Pump thrombosis and replacement	186 009 (54 547-497 289) [37 202]	γ	Personal communication (email from KL Kerk, RN, BN, National Heart Centre Singapore, November 18, 2022, and February 28, 2023)
Worsening HF	9501 (3810-9667) [903]	γ	Malhotra et al,^25^ 2018
Routine care for LVAD, cost per mo	436 (249-679) [87]	γ	Personal communication (email from KL Kerk, RN, BN, National Heart Centre Singapore, November 18, 2022, and February 28, 2023)
Routine care for MM inotrope dependent, cost per mo	6691 (249-13 082) [1338]	γ	Personal communication (email from KL Kerk, RN, BN, National Heart Centre Singapore, November 18, 2022, and February 28, 2023); Malhotra et al,^25^ 2018
Routine care for MM inotrope independent, cost per mo	230 (43-474) [46]	γ	Personal communication (email from KL Kerk, RN, BN, National Heart Centre Singapore, November 18, 2022, and February 28, 2023)
In-hospital death	41 268 (5359-101 451) [6455]	γ	Malhotra et al,^25^ 2018

Abbreviations: AE, adverse event; ESHF, end-stage heart failure; HF, heart failure; HR, hazard ratio; LVAD, left ventricular assist device; MM, optimal medical management; NV, not varied; RVAD, right ventricular assist device; SGD, Singapore dollars.

^a^ We assumed that pump replacement occurs in all patients who experience thrombosis.

^b^ Hospitalizations due to worsening HF were derived by subtracting events per patient-year for all other AEs from the events per patient-year for all-cause rehospitalization.

^c^ To convert SGD to US dollars, multiply by 0.75.

**Figure 1.**
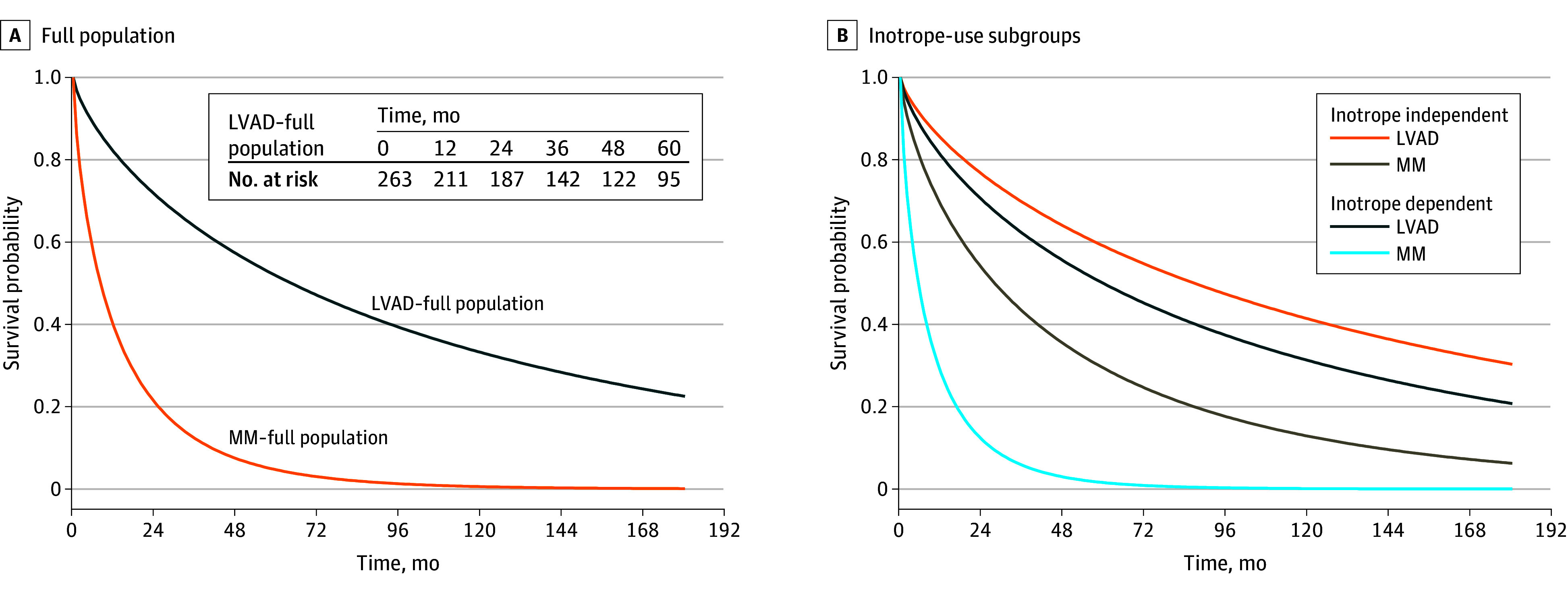
Estimated Survival for Transplant-Ineligible Patients A, Survival with left ventricular assist device (LVAD) was estimated for the full destination therapy (DT) population using mortality data from the MOMENTUM 3 (Multicenter Study of MagLev Technology in Patients Undergoing Mechanical Circulatory Support Therapy With HeartMate 3) trial that were age-adjusted for the Singaporean population and extrapolated to 15 years. Survival with optimal medical management (MM) was estimated for the full DT population using an all-cause mortality hazard ratio of MM vs LVAD derived from MOMENTUM 3 and prior trial results. B, Survival according to inotrope use was derived using survival for the full DT population, subgroup analyses from the MOMENTUM 3 and REMATCH (Randomized Evaluation of Mechanical Assistance for the Treatment of Congestive Heart Failure) trials, and ROADMAP (Risk Assessment and Comparative Effectiveness of LVAD and MM in Ambulatory Heart Failure) trial results (eFigure 2 in Supplement 1). See eAppendix in Supplement 1.

Beginning with LVAD, we estimated survival for the transplant-ineligible population using mortality data from the MOMENTUM 3 (Multicenter Study of MagLev Technology in Patients Undergoing Mechanical Circulatory Support Therapy with HM 3) trial (Figure 1; eAppendix in Supplement 1). We digitally extracted data from the published 5-year DT survival curve, fit multiple survival distributions to the data, and selected a Weibull distribution. Using the Weibull curve parameters, we extrapolated survival to 15 years and then age-adjusted it using Singapore Life Tables.^26^ We estimated MM survival using a hazard ratio (HR) derived as the product of all-cause mortality HRs reported in trials comparing MM vs the first-generation device, the first-generation device vs second-generation LVAD, and the second-generation vs current LVAD. We multiplied this HR by monthly death rates for LVAD to estimate corresponding monthly death rates for MM, which we transformed to probabilities. We differentiated survival according to inotrope use, applying similar steps and subgroup-specific HRs (eFigure 2 in Supplement 1).

### AEs and Heart Failure Hospitalizations

We derived monthly probabilities of stroke, poststroke mortality, and AEs other than stroke from peer-reviewed literature (Table 1).^21^ Compared with stroke, all other AEs experienced by patients are generally less severe and resolve more quickly; thus, we assumed they would resolve within the cycle in which they occurred.

### Utilities

We estimated QALYs using health state utilities (HSUs) that range from 0 (indicating death) to 1 (indicating perfect health). We estimated HSUs for patients who were alive without stroke as a weighted mean of utilities associated with NYHA symptom classes (Table 1).^22^

Patients who were alive after stroke were assigned an HSU specific to their stroke severity.^23^ We consulted peer-reviewed literature to determine the proportion of patients who experienced disabling vs nondisabling stroke and estimated corresponding weighted means of these utilities.^20,24^ For short-term AEs, including heart failure hospitalizations, we applied a 10% decrement to the HSU within the cycle in which the AE occurred. HSUs and AE decrements were modeled until death when patients were assigned a utility of 0.

### Costs

We estimated costs using a health care system perspective, in accordance with Singapore’s Agency for Care Effectiveness guidelines.^27^ We obtained LVAD cost estimates from the National Heart Centre Singapore (KL Kerk, RN, BN, email, November 18, 2022, and February 28, 2023), which runs the largest mechanical circulatory support program in the country (Table 1). We estimated additional costs relevant to both treatment groups using billing data from the Singapore Cohort of Patients with Advanced Heart Failure study, which enrolled 276 patients with NYHA class III and IV symptoms between July 2017 and August 2019 and follows participants until death.^25^ Annual medical record extractions include diagnosis codes that can be linked to accident and emergency, inpatient, outpatient, and pharmacy billing data (eTable 1 in Supplement 1). In Singapore, facility and physician medical bills for nonsubsidized patients are set to recover mean costs, and we estimated mean costs using these charges. Costs are reported in 2023 Singapore dollars (SGD).

Consistent with recommended practice, we discounted costs and QALYs by 3% annually.^27^ We consulted with clinicians to confirm the model’s face validity.

### Base Case Analysis

ICERs were calculated as the mean incremental cost divided by the mean incremental QALYs of LVAD compared with MM. In the base case, we estimated an ICER for the transplant-ineligible population by modeling outcomes for inotrope-dependent and inotrope-independent subgroups.

Because the Agency for Care Effectiveness does not specify a cost-effectiveness threshold, we followed the American College of Cardiology and American Heart Association recommendations, which are consistent with historic World Health Organization guidelines. These recommendations designate an intervention as cost-effective (ie, high value) if the ICER is less than 1 times the gross domestic product (GDP) per capita (ie, SGD 114 000 [US $85 075]), as intermediate value if the ICER is between 1 and less than 3 times the GDP per capita, and as low value if the ICER is equal to or greater than 3 times the GDP per capita.^28^

### Sensitivity, Scenario, and Threshold Analyses

We accounted for statistical uncertainty underlying model inputs through probabilistic sensitivity analysis. We assigned distributions to inputs according to parameter type and ran 1000 Monte Carlo simulations (Oracle Crystal Ball, version 11.1; Oracle). We also investigated the robustness of our base case results when replacing inputs with low-end and high-end estimates derived from the literature or estimation in 1-way deterministic sensitivity analyses.

To investigate impacts of alternative structural assumptions, we performed 3 scenario analyses. First, we used data from the National Heart Centre Singapore (personal communication from KL Kerk, RN, BN, email, July 8, 2022) to estimate DT LVAD survival and AE probabilities (eTable 2 in Supplement 1). Although the National Heart Centre Singapore patient population remains small (n = 36), it provided data that constitute clinical evidence for Singapore. Second, we investigated how the results would be affected if maximum survival was 10 years, as opposed to 15 years in the base case. While LVAD survival beyond 10 years has been observed and could eventually be achieved in Singapore given that health care outcomes are often above average, the longest observed duration that a patient has had DT LVAD support is 9 years, according to KL Kerk, RN, BN (email, July 8, 2022). Third, we investigated how the results would be affected if we did not differentiate outcomes by inotrope use but if we instead used mean survival and cost estimates for the full population.

We performed threshold analyses to estimate the extent to which the total implantation (ie, device plus procedure) cost, AE costs, and LVAD survival benefit would have to change to yield an ICER less than SGD 114 000 (US $85 075) per QALY gained. We investigated the extent to which individual inputs would have to change and determined combinations of total implantation costs and all-cause mortality HRs that would yield ICERs less than SGD 114 000 per QALY gained.

### Statistical Analysis

Statistical analyses were performed from December 2023 to July 2024 using Stata, version 18 (StataCorp LLC). To select a survival distribution, we compared exponential, Gompertz, log-logistic, and Weibull regression results using log likelihoods, Akaike information criteria, and bayesian information criteria. We also performed a likelihood ratio (LR) test to evaluate Weibull fit compared with the exponential distribution, which is often used to estimate heart failure survival (LR = 14.17, *P* < .001 for base case regressions using trial data; LR = 13.3, *P* < .001 for base case regressions using local survival data). Two-sided *P* < .05 indicated statistical significance.

Analyses of cohort billing data were descriptive (ie, mean cost estimation). Locally observed AE rates used in a scenario analysis were estimated as events per patient-year.

## Results

**Figure 2.**
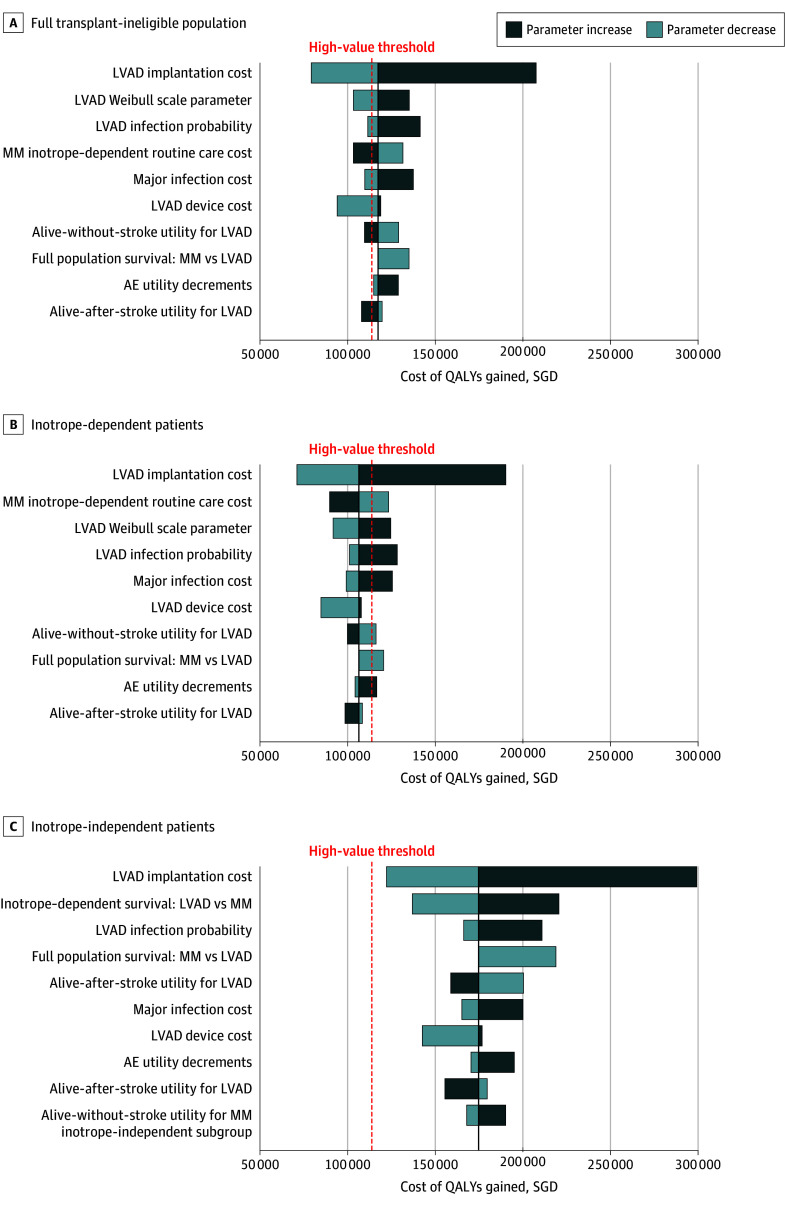
Incremental Cost-Effectiveness Ratios (ICERs) When Replacing Single Inputs With Low-End and High-End Estimates The 10 most influential inputs into the ICER are shown for full transplant-ineligible population, inotrope-dependent patients, and inotrope-independent patients. AE indicates adverse event; LVAD, left ventricular assist device; MM, optimal medical management; QALYs, quality-adjusted life-years; and SGD, Singapore dollars. To convert SGD to US dollars, multiply by 0.75.

At model initiation, the cohort had a mean (SD) age of 64 (12) years, and 78% (range, 68%-86%) of patients were inotrope dependent (Table 1). In the base case analysis, patients implanted with LVAD lived a mean 5.95 years and experienced 4.15 (95% uncertainty interval [UI], 1.90-6.95) QALYs at a cost of SGD 496 969 (95% UI, SGD 391 903-627 302 [US $370 872; 95% UI, $292 465-$468 136]). Patients who received MM lived a mean 1.29 years and experienced 0.71 (95% UI, 0.32-1.65) QALYs at a cost of SGD 92 292 (95% UI, SGD 51 038-209 030 [US $68 875; 95% UI, $38 088-$155 993]). These outcomes resulted in an incremental gain of 3.45 QALYs at an incremental cost of SGD 404 678 (US $301 999), producing an ICER of SGD 117 370 (95% UI, SGD 62 110-304 198 [US $87 590; 95% UI, $46 351-$227 013]) per QALY gained (Table 2), indicating that LVAD is an intermediate value intervention for the transplant-ineligible population (eFigure 5 in Supplement 1). In probabilistic sensitivity analysis, 50% of ICER replications were below the threshold of SGD 114 000 (US $85 075) per QALY gained (eFigures 3 and 4 in Supplement 1). When replacing model inputs with end-of-range estimates in deterministic sensitivity analyses, the ICERs ranged from SGD 79 238 to 207 648 (US $59 133-$154 961) per QALY gained (Figure 2).

**Table 2.** Costs, Effectiveness, and Incremental Cost-Effectiveness of LVAD and MM^a^

Treatment	Patients, mean (SD)					
	Full population (N = 100)		Inotrope dependent (n = 78)		Inotrope independent (n = 22)	
	LVAD	MM	LVAD	MM	LVAD	MM
Base case, mean (2.5-97.5 percentiles)						
Total per-person costs, SGD	496 969 (391 913-627 280)	92 292 (51 038-209 030)	496 152 (389 737-621 604)	100 711 (52 713-241 464)	499 865 (379 536-648 998)	62 441 (35 037-111 539)
QALYs	4.15 (1.90-6.95)	0.71 (0.32-1.65)	4.13 (1.72-6.91)	0.41 (0.18-1.17)	4.24 (1.51-7.81)	1.74 (0.69-3.69)
ICER, SGD per QALY gained	117 370 (62 110-304 198)	NA	106 458 (52 398-262 870)	NA	174 798 (150 653-1 105 292)	NA
Scenario analyses						
LVAD survival estimation with clinical data						
Total per-person costs, SGD	479 567	68 110	480 996	69 615	472 586	60 762
QALYs	3.97	0.50	3.91	0.26	4.25	1.68
ICER, SGD per QALY gained	118 711	NA	112 704	NA	160 410	NA
10-y Stop and drop						
Total per-person costs, SGD	475 507	91 530	474 955	100 588	477 465	59 415
QALYs	3.49	0.68	3.47	0.41	3.55	1.63
ICER, SGD per QALY gained	136 603	NA	122 316	NA	217 123	NA
Model without inotrope-use differentiation						
Total per-person costs, SGD	497 337	122 420	NA	NA	NA	NA
QALYs	4.15	0.66				
ICER, SGD per QALY gained	107 444	NA				

Abbreviations: ICER, incremental cost-effectiveness ratio; LVAD, left ventricular assist device; MM, optimal medical management; NA, not applicable; QALYs, quality-adjusted life-years; SGD, Singapore dollars.

^a^ To convert SGD to US dollars, multiply by 0.75.

For patients with inotrope dependency at the time of implantation, LVAD produced a relatively greater incremental gain in QALYs (3.71) at lower incremental cost (SGD 395 442 [US $295 106]), yielding a high-value ICER of SGD 106 458 (US $79 446) per QALY gained. For inotrope-independent patients, a smaller incremental QALY gain (2.50) at higher incremental cost (SGD 437 424 [US $326 436]) yielded an ICER of SGD 174 798 (US $130 446) per QALY gained. In probabilistic sensitivity analysis, 59% and 19% of replications were below the high-value threshold for inotrope-dependent and inotrope-independent patients, respectively. In deterministic sensitivity analyses, the inotrope-dependent ICER ranged from SGD 71 063 to 190 255 (US $53 032-$141 981) per QALY gained, and the inotrope-independent ICER ranged from SGD 122 260 to 299 183 (US $91 239-$223 271) per QALY gained. ICERs for both subgroups were affected by implantation procedure and device costs, LVAD infection probability, and infection cost. For inotrope-dependent patients, the cost of MM routine care was also highly influential. For inotrope-independent patients, LVAD survival compared with MM was highly influential.

### Scenario and Threshold Analyses

Estimating LVAD survival using clinical data from Singapore rather than age-adjusted survival curves from the MOMENTUM 3 trial increased the ICER to SGD 118 711 (US $88 590) per QALY gained (Table 2). When assuming survival up to 10 years, rather than 15 years, the ICER increased to SGD 136 603 (US $101 943) per QALY gained. Constructing a model with mean inputs for the DT population without differentiating inotrope-use status produced an ICER of SGD 107 444 (US $80 182) per QALY gained.

**Figure 3.**
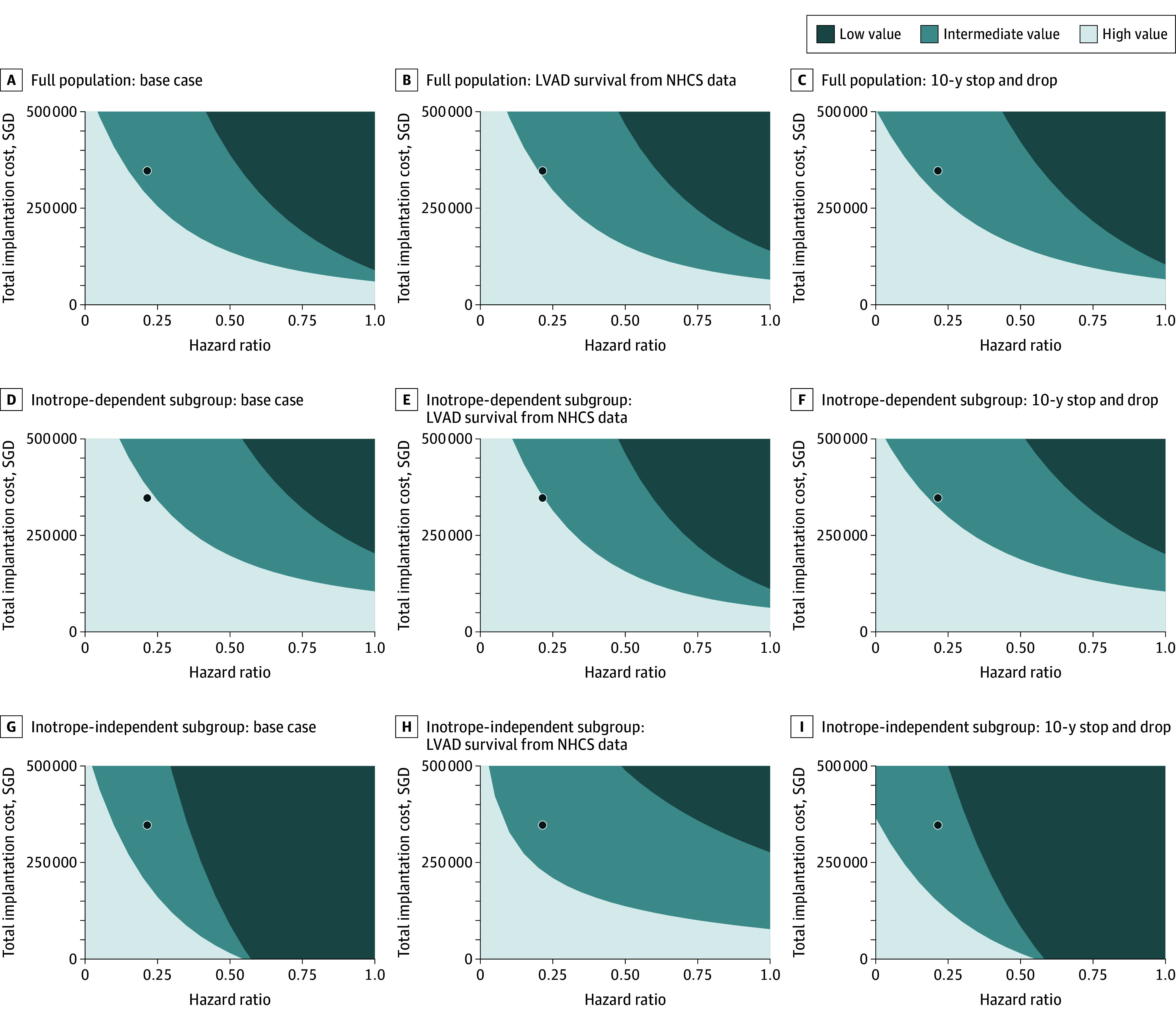
Cost-Effectiveness According to Left Ventricular Assist Device (LVAD) Hazard Ratio (HR) and Total Implantation Cost Shaded areas represent input combinations resulting in high-, intermediate-, and low-value interventions. The small circle represents the incremental cost-effectiveness ratio for each population and scenario. NHCS indicates National Heart Centre Singapore; and SGD, Singapore dollars. To convert SGD to US dollars, multiply by 0.75.

Threshold analyses revealed that the total implantation cost, lifetime AE costs, or all-cause mortality HR of LVAD vs MM would have to be reduced by 3%, 11%, or 5%, respectively, to yield a high-value ICER for the full population. For inotrope-independent patients, a 44% decrease in the total implantation cost or a 54% decrease in the all-cause mortality HR would be needed to yield a high-value ICER. Lifetime AE costs could not be sufficiently reduced to achieve a high-value ICER for this subgroup. The probability that the inotrope-dependent ICER is high value could be increased to 75%, 85%, and 95% with 20%, 33%, and 54% decreases in the total implantation cost, respectively. Combinations of total implantation costs and all-cause mortality HRs that would yield a high-value ICER for the full patient population and inotrope-use subgroups are presented in Figure 3.

## Discussion

To our knowledge, these results constitute the first economic evaluation evidence of LVAD for ESHF in Singapore. We found that LVAD returned greater quality-adjusted survival and costs than MM and is likely an intermediate-value intervention for the transplant-ineligible population with an ICER of SGD 117 370 (US $87 590) per QALY gained. Yet, we also found that value differed according to inotrope use. The ICER was considerably lower and was high value (SGD 106 458 [US $79 446] per QALY gained) for inotrope-dependent patients who formed most of the transplant-ineligible population (68%-86% of trial participants and approximately 80% of local patients), whereas substantial price reductions and/or survival increases would be required to decrease the inotrope-independent ICER (SGD 174 798 [US $130 446] per QALY gained) to the high-value threshold.

Probabilistic sensitivity analysis confirmed that LVAD is likely to be high value for the inotrope-dependent population, yet statistical uncertainty led to a conclusion of intermediate value in a substantial proportion (41%) of cases. Additionally, the inotrope-dependent ICER became intermediate value when we used several end-of-range input estimates and in a scenario where we limited survival to 10 years. Although the 10-year ICER might better approximate LVAD’s value as thus far observed, the base case ICER likely better approximates LVAD’s value at scale given that total volume of implantations and observable duration of follow-up in Singapore have been limited. Sensitivity and scenario analyses consistently showed that LVAD is unlikely to be high value for inotrope-independent patients yet remains in the intermediate-value range.

Given the evidence of high value for inotrope-dependent patients and the uncertainty concerning this result, decision-makers could consider prioritizing LVAD for inotrope-dependent patients while encouraging plausible price reductions (eg, 20%-33% of total implantation price) and/or technological advancements to more definitively establish LVAD’s cost-effectiveness. The results do not support subsidization for inotrope-independent patients.

Centrifugal flow devices have been the focus of cost-effectiveness analyses, including 2 studies that compared the HeartWare (Medtronic) ventricular assist device (VAD) with MM in the US and UK.^29,30^ This VAD was smaller and required a less invasive implantation procedure, yet a series of defects led to its market withdrawal in 2021. Although the ICER that we estimated is higher than those ICERs estimated for the VAD (eFigure 4 in Supplement 1), the VAD studies did not account for device defects and the ICERs were underestimated. Additionally, a study has investigated LVAD compared with MM for transplant-ineligible patients in the UK.^15^ The study reported that LVAD was not cost-effective at a conventional £30 000 threshold (ie, slightly less than 1 times the national GDP per capita), yet the ICER was below an elevated threshold for end-of-life interventions (ie, £50 000 per QALY gained). However, the UK has since replaced the end-of-life premium with a severity modifier that is harder to achieve and less generous.^31^ Our conclusion that LVAD is an intermediate-value intervention for the full transplant-ineligible population is consistent with the UK study results, yet the ICER we calculated more closely approaches GDP per capita, and we found that LVAD attains high value at a conventional threshold for a majority of patients who are inotrope dependent.

### Strengths and Limitations

This study’s strengths include the modeling of costs and outcomes by inotrope use. A lower ICER calculated in a scenario analysis using mean population estimates underscores the importance of directly modeling subgroup differences. We localized the analysis using age-adjusted survival and high-quality clinical cost estimates. We also made novel use of local observational survival evidence in a scenario analysis and demonstrated methodological rigor by partitioning poststroke survival. The results are important as they can be used to inform a subsidy determination. To our knowledge, the results are the first to indicate that LVAD achieves cost-effectiveness at a conventional threshold for the majority subgroup of patients, and threshold analyses of changes that can be made to reduce uncertainty underlying this result are informative.

This study has limitations. First, we estimated survival using mortality data from randomized clinical trials conducted in North America.^32^ Although we accounted for population age differences, factors beyond age could contribute to ESHF survival differences in Singapore.^33,34^ Extrapolating survival using observed mortality for patients receiving LVAD in Singapore led to the same conclusion that LVAD is, generally, high value for inotrope-dependent patients and intermediate value for inotrope-independent patients. Yet, LVAD outcomes should continue to be monitored for additional patients and over longer duration. Second, the MM survival estimation required the use of results from the REMATCH (Randomized Evaluation of Mechanical Assistance for the Treatment of Congestive Heart Failure) trial that was conducted over 2 decades ago.^6^ Since the REMATCH trial, medical therapy improvements have largely been limited to earlier-stage disease (although few indications have recently been extended to patients with NYHA class IIIB symptoms), and our approach arguably accounted for secular time patterns that were the same for LVAD and MM. Yet, contemporary investigation of MM survival using experimental or quasiexperimental (eg, propensity score) methods with expanding observational evidence could validate the present study’s results.^35^ Third, we estimated survival for inotrope-use subgroups yet subgroup-specific AE probabilities and LVAD symptom improvements were inconsistently reported in prior trials, necessitating assumptions based on data for the full population. AE probabilities and utilities were not the most influential model inputs, yet the results could be improved with subgroup-specific data for these inputs. Fourth, while we reported results by inotrope use, whether social dimensions substantially affect LVAD and MM outcomes is not thoroughly understood (in Singapore or globally), and this study does not evaluate any equity impacts of treatment.

## Conclusions

In this economic evaluation, LVAD was found to be potentially high value for transplant-ineligible patients who are inotrope dependent. Confidence in this result was improved with modest price reductions or technological advancements, but only extreme changes rendered LVAD high value for inotrope-independent patients.

## Notes

Supplement 1. **eFigure 1.** Health State Transition Diagram **eFigure 2.** Survival Estimation for Inotrope Use Subgroups **eTable 1.** Diagnosis Codes Used in Cost Estimation **eTable 2.** National Heart Centre Singapore Patient Characteristics **eFigure 3.** Cost Effectiveness Plane of HM3 LVAD Versus MM **eFigure 4.** Cost-Effectiveness Acceptability of HM3 LVAD Versus MM **eFigure 5.** Global ICER Estimates of DT LVAD Versus MM **eAppendix.** Figure 1 No. at Risk Explanation and Monthly Survival Probabilities
eReferences

Supplement 2.
Data Sharing Statement
