# Supplementary material for: Cost-Effectiveness of Left Ventricular Assist Device for Transplant-Ineligible Patients
Source: JAMA Netw Open. 2025 Apr 18;8(4):e254483. doi: 10.1001/jamanetworkopen.2025.4483 (PMC12008763; doi:10.1001/jamanetworkopen.2025.4483)
Supplement: Supplement 1. — eFigure 1. Health State Transition Diagram eFigure 2. Survival Estimation for Inotrope Use Subgroups eTable 1. Diagnosis Codes Used in Cost Estimation eTable 2. National Heart Centre Singapore Patient Characteristics eFigure 3. Cost Effectiveness Plane of HM3 LVAD Versus MM eFigure 4. Cost-Effectiveness Acceptability of HM3 LVAD Versus MM eFigure 5. Global ICER Estimates of DT LVAD Versus MM eAppendix. Figure 1 No. at Risk Explanation and Monthly Survival Probabilities eReferences [file jamanetwopen-e254483-s001.pdf]

Supplemental Online Content

Schaffer EM, Su RGS, Chay J, Finkelstein EA. Cost-effectiveness of left ventricular assist device for transplant-ineligible patients. *JAMA Netw Open*. Published online April 10, 2025. doi:10.1001/jamanetworkopen.2025.4483

eFigure 1. Health State Transition Diagram

eFigure 2. Survival Estimation for Inotrope Use Subgroups

eTable 1. Diagnosis Codes Used in Cost Estimation

eTable 2. National Heart Centre Singapore Patient Characteristics

eFigure 3. Cost Effectiveness Plane of HM3 LVAD versus MM

eFigure 4. Cost-Effectiveness Acceptability of HM3 LVAD versus MM

eFigure 5. Global ICER Estimates of DT LVAD versus MM

eAppendix. Figure 1 No. at Risk Explanation and Monthly Survival Probabilities

eReferences

This supplemental material has been provided by the authors to give readers additional information about their work.

© 2025 Schaffer EM et al. *JAMA Network Open*

eFigure 1. Health State Transition Diagram

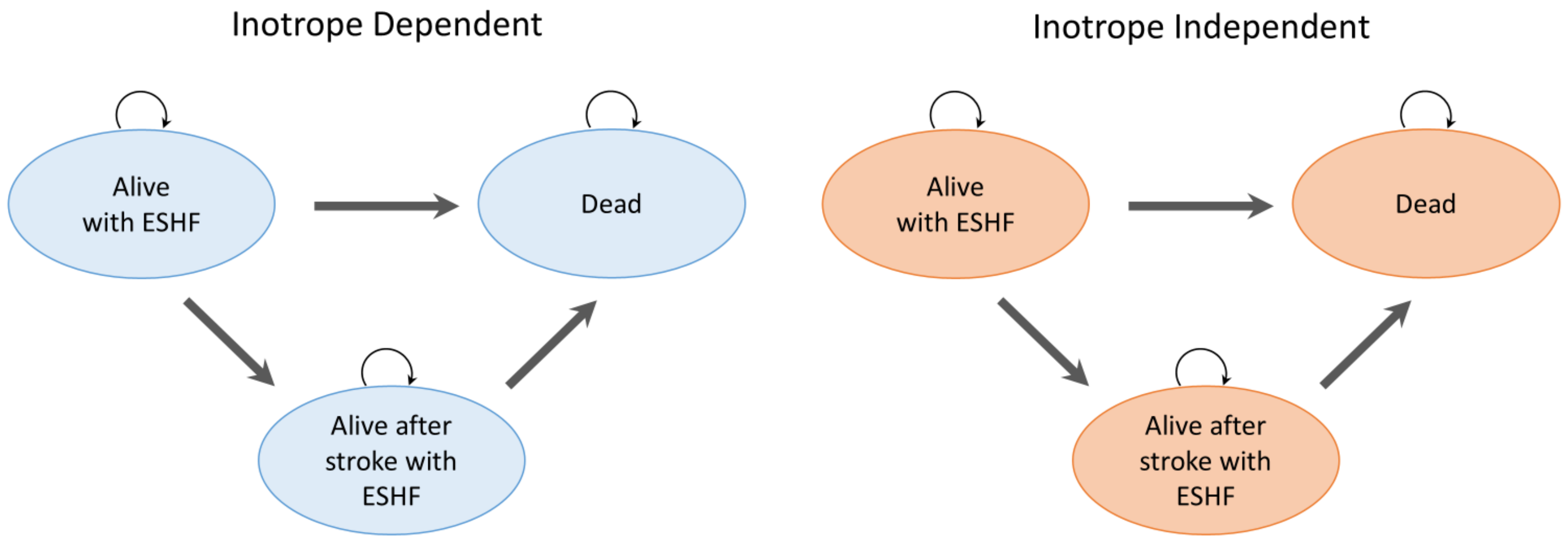

All patients enter the Markov model in the "Alive with end-stage heart failure (ESHF)" state and can remain in the state or transition to the "Alive after stroke with ESHF" or "Dead" states. Patients in the "Alive after stroke with ESHF" can remain in the state or transition to the "Dead" state. Patients in each of the alive states experience probabilities of minor adverse events and of stroke (i.e., initial or recurrent) each cycle. Once patients transition to the "Dead" state, they remain in that state for the duration of the model. Health state transitions were modeled separately according to inotrope dependence, and we assumed that patients who were inotrope-independent did not transition to dependent (and vice versa) over the model duration.

© 2025 Schaffer EM et al. *JAMA Network Open*

eFigure 2. Survival Estimation for Inotrope Use Subgroups

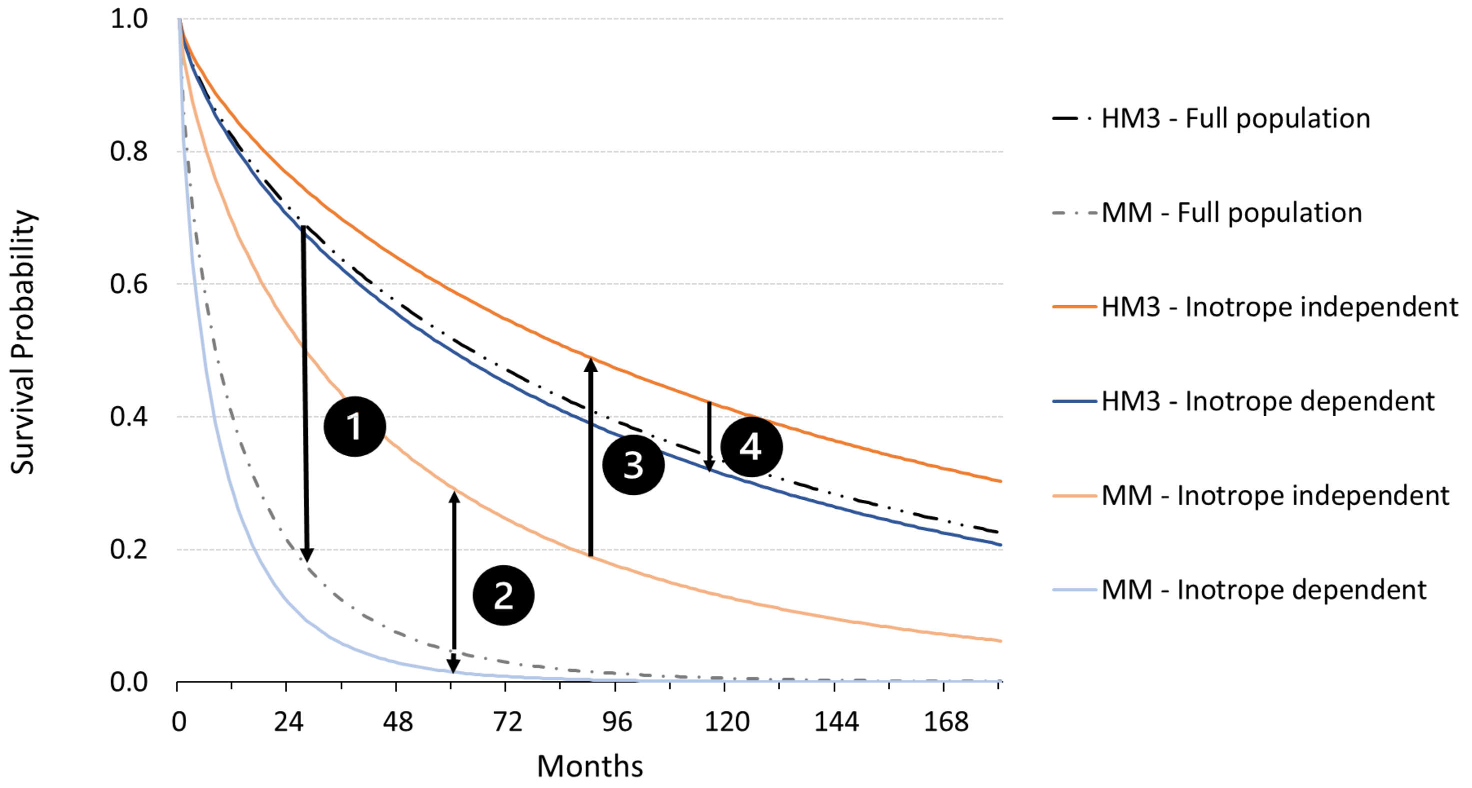

We estimated survival according to inotrope-use as follows: 1) Estimated MM survival relative to HM3 LVAD using an all-cause mortality HR calculated from trials comparing MM to HM XVE, XVE to HM2, and HM2 to HM3; 2) Estimated MM survival for inotrope-dependent and -independent patients using subgroup analyses from REMATCH trial[1]; 3) Estimated HM3 survival for inotrope-independent patients using ROADMAP study[2] and subgroups results from MOMENTUM 3 trial[3]; 4) Derived HM3 survival for inotrope-dependent patients using inotrope-independent survival (estimated in step 3) and HM3 survival for the full population.

Abbreviations: HM3, HeartMate 3 left ventricular assist device; MM, optimal medical management; REMATCH, Randomized Evaluation of Mechanical Assistance for the Treatment of Congestive Heart Failure; ROADMAP, Risk Assessment and Comparative Effectiveness of LVAD and MM in Ambulatory Heart Failure; MOMENTUM 3, Multicenter Study of MagLev Technology in Patients Undergoing Mechanical Circulatory Support Therapy with HM 3.

© 2025 Schaffer EM et al. *JAMA Network Open*

eTable 1. Diagnosis Codes Used in Cost Estimation

Using data from the Singapore Cohort of Patients with Advanced Heart Failure (SCOPAH)[4], we used ICD10 codes to identify cases for the following conditions. We then used these case numbers identify accident and emergency (A&E), inpatient, and pharmacy billing data to estimate adverse event costs for patients who are receiving medical management. We cannot tease out costs due to secondary diagnoses and thus assign all costs for a case to the primary diagnosis code.

Adverse events and care	ICD10 codes and free text search terms used
Stroke	I60, I61, I63, I64
Major infection	A41, T82.7, T81.4
Bleeding	K92.2, R58
Worsening HF	I50.0, I50.1, I50.9
Routine care for MM – Inotrope dependent	We searched for “dobutamine” and “milrinone” in inpatient and pharmacy billing data.
In-hospital death	Identified patients who died in hospital using search terms “Death”, “Death – Coroner Case”.

© 2025 Schaffer EM et al. *JAMA Network Open*

eTable 2. National Heart Centre Singapore Patient Characteristics

Characteristics (Number (%) unless otherwise specified)	Full Population (n=36)	Inotrope-Dependent (n=30)	Inotrope-Independent (n=6)
**Baseline characteristics**			
Age, years (mean, SD)	64.4(4.5)	63.8(3.7)	67.5(7.2)
Male	29(81%)	25(83%)	4(67%)
Female	7(19%)	5(17%)	2(33%)
**Index hospitalization characteristics**			
Intensive care unit length of stay, days (median, IQR)	8.0(5.0-24.5)	8.0(5.0-27.0)	8.0(5.0-13.0)
Total hospital length of stay, days (median, IQR)	33.0(24.0-55.5)	34.5(25.0-51.0)	30.0(21.0-60.0)
Use of right ventricular assist device	4(11%)	3(10%)	1(17%)
Mortality	8(22%)	8(27%)	0(0%)
**Survival estimation**[a]			
Weibull scale parameter	0.0842	0.1114	0.0032×10^{-3}
Weibull shape parameter	0.5054	0.4650	2.7924
**Events per patient-year**[a]			
Stroke	0.1064	0.1380	0.0000
Major infection	0.2210	0.2123	0.2500
Bleeding	0.3274	0.3716	0.1786
Right heart failure - Not managed w/RVAS	0.0082	0.0106	0.0000
Pump thrombosis	0.0164	0.0212	0.0000
Heart failure decompensation or other hospitalization	0.7284	0.6477	1.0000
Hospital readmission (any cause)[b]	1.4528	1.4512	1.4643

[a]Excludes data from one patient with an incorrect death date.
[b]Calculated for patients who survived the index admission.

© 2025 Schaffer EM et al. *JAMA Network Open*

eFigure 3. Cost-Effectiveness Plane of HM3 LVAD versus MM

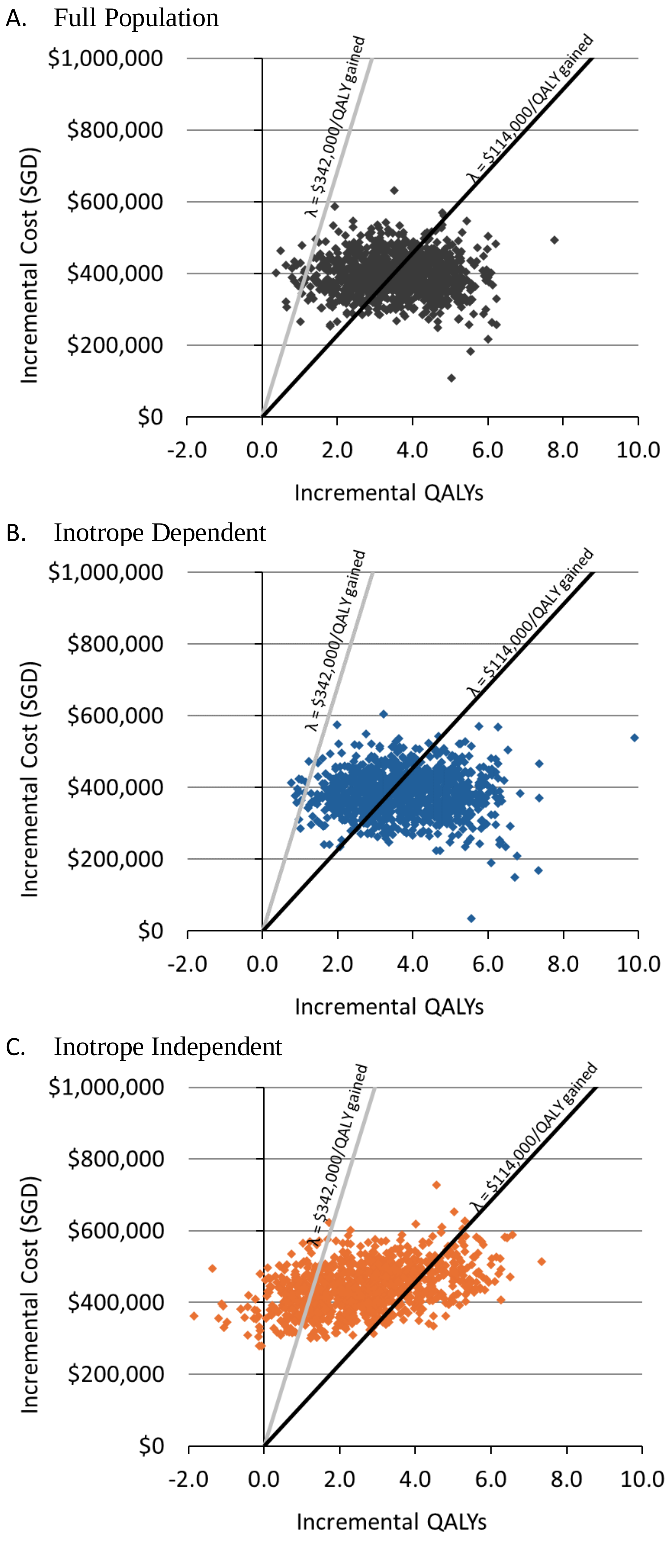

Each point represents the incremental quality-adjusted life-years (QALYs) and cost estimated for a single random draw of model inputs, taking into account input statistical uncertainty. Points that fall below the black line indicate a high-value intervention. Those that fall between the grey and black lines indicate an intermediate-value intervention. Abbreviations: HM3 LVAD, HeartMate 3 left ventricular assist device; MM, optimal medical management; λ, willingness-to-pay threshold; SGD, Singapore dollars.

© 2025 Schaffer EM et al. *JAMA Network Open*

eFigure 4. Cost-Effectiveness Acceptability of HM3 LVAD versus MM

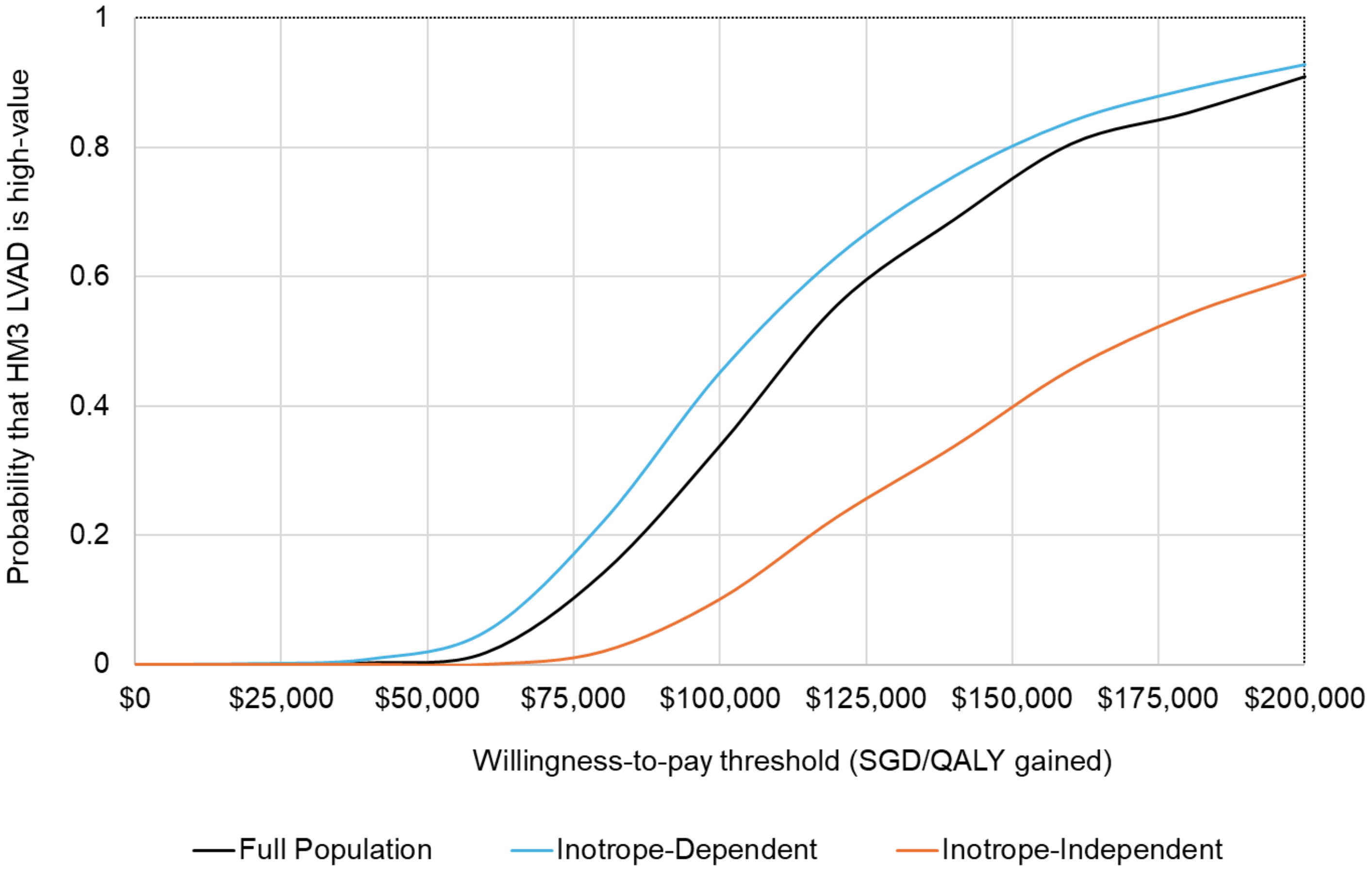

The cost-effectiveness acceptability curve indicates the probability that HM3 LVAD is high-value relative to MM over a range of willingness-to-pay thresholds. Abbreviations: HM3 LVAD, HeartMate 3 left ventricular assist device; MM, optimal medical management; SGD, Singapore dollars; QALY, quality-adjusted life-year

© 2025 Schaffer EM et al. *JAMA Network Open*

eFigure 5. Global ICER Estimates of DT LVAD versus MM

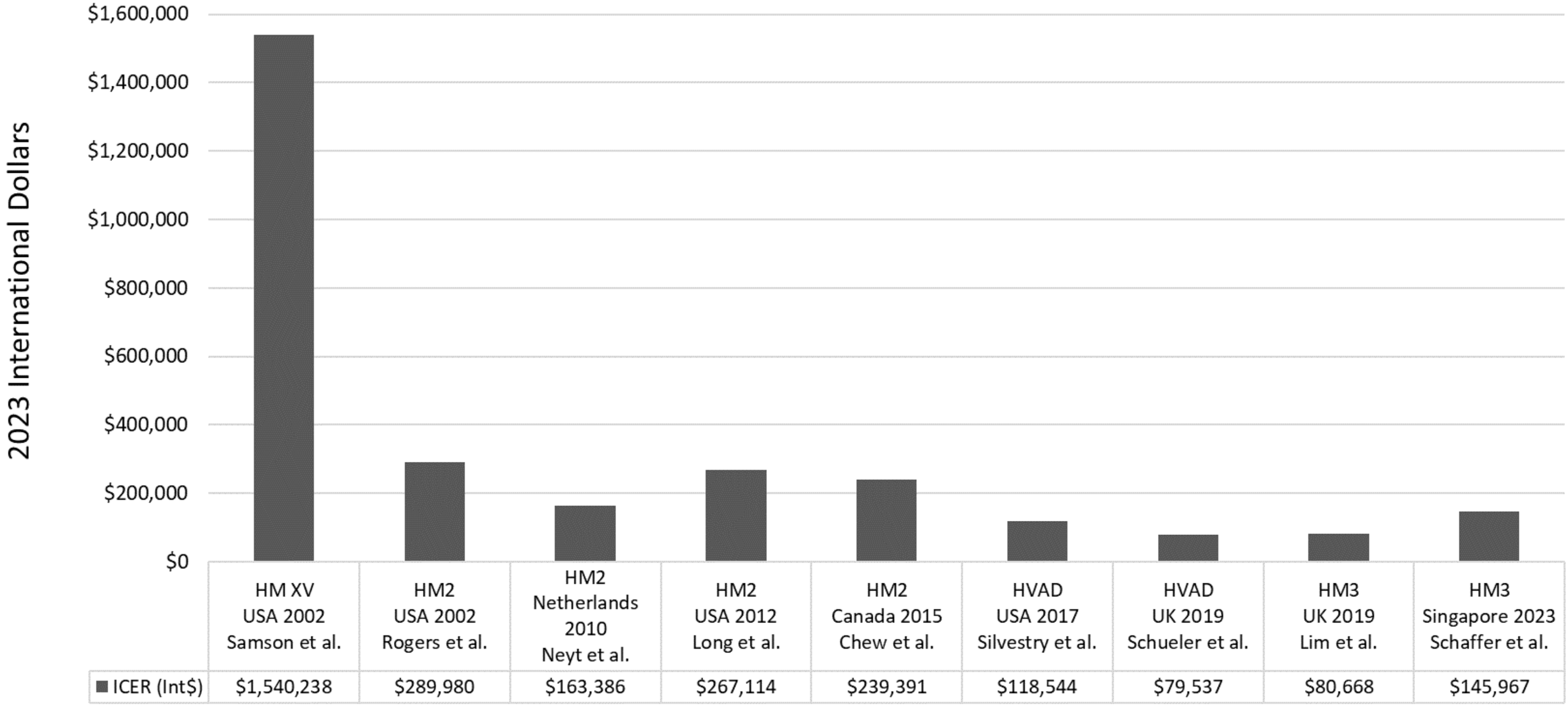

	HM XV USA 2002 Samson et al.	HM2 USA 2002 Rogers et al.	HM2 Netherlands 2010 Neyt et al.	HM2 USA 2012 Long et al.	HM2 Canada 2015 Chew et al.	HVAD USA 2017 Silvestry et al.	HVAD UK 2019 Schueler et al.	HM3 UK 2019 Lim et al.	HM3 Singapore 2023 Schaffer et al.
ICER (Int$)	$1,540,238	$289,980	$163,386	$267,114	$239,391	$118,544	$79,537	$80,668	$145,967

ICERs were inflated to 2023 values and converted to international dollars (Int$) to enable cross-country comparison. Abbreviations: ICER, incremental cost effectiveness ratio; DT LVAD, destination therapy left ventricular assist device; MM, optimal medical management; HM, HeartMate.[5-12]

© 2025 Schaffer EM et al. *JAMA Network Open*

eAppendix. Figure 1 No. at Risk Explanation and Monthly Survival Probabilities

In Figure 1A, no. at risk is provided for the LVAD—full population survival curve up to month 60, in accordance with 5-year mortality data that have been published from the MOMENTUM 3 trial. No. at risk is not reported for the MM—full population survival curve as MM survival was estimated from an indirect comparison, which used results from multiple trials sharing referent comparators and is recommended practice in the absence of a direct, head-to-head comparison. Reporting of no. at risk for MM is thus not informative in the way no. of risk typically is (accounting for censoring), and we think employing a meta-analysis or an assumption to indicate no. at risk for MM is likely to be confusing and/or misleading. For these same reasons, we have not included no. at risk for the survival curves in Figure 1B.

Following are tables indicating expected survival probabilities by monthly time increments for each treatment group and subgroup represented in Figure 1. These tables provide similar information without the challenges involved in reporting no. at risk.

		**Full Population**		**Inotrope-Use Subgroups**			
	HM3 - Full Population From 5-yr MOMENTUM 3 trial outcomes, with extrapolation	HM3- Full Population From 5-yr MOMENTUM 3 trial outcomes with extrapolation and age-adjusted for Singapore population	MM - Full Population Derived via indirect comparison and age-adjusted for Singapore population	HM3 - Inotrope-Independent Derived via indirect comparison and age-adjusted for Singapore population	HM3 - Inotrope-Dependent Derived via indirect comparison and age-adjusted for Singapore population	MM - Inotrope-Independent Derived via indirect comparison and age-adjusted for Singapore population	MM - Inotrope-Dependent Derived via indirect comparison and age-adjusted for Singapore population
Time (mo.)	S(t)	S(t)	S(t)	S(t)	S(t)	S(t)	S(t)
0	1	1	1	1	1	1	1
1	0.9688	0.9687	0.8625	0.9749	0.9670	0.9426	0.8177
2	0.9484	0.9483	0.7811	0.9583	0.9455	0.9059	0.7145
3	0.9310	0.9309	0.7163	0.9442	0.9271	0.8751	0.6351
4	0.9154	0.9151	0.6617	0.9314	0.9106	0.8478	0.5702
5	0.9010	0.9007	0.6144	0.9196	0.8954	0.8230	0.5154
6	0.8875	0.8871	0.5726	0.9085	0.8812	0.8001	0.4683
7	0.8748	0.8744	0.5352	0.8980	0.8678	0.7788	0.4273
8	0.8627	0.8623	0.5016	0.8880	0.8551	0.7588	0.3911
9	0.8512	0.8507	0.4710	0.8784	0.8430	0.7399	0.3590
10	0.8402	0.8396	0.4430	0.8692	0.8314	0.7221	0.3304
11	0.8295	0.8289	0.4174	0.8603	0.8203	0.7051	0.3046
12	0.8193	0.8186	0.3938	0.8518	0.8095	0.6888	0.2814
13	0.8094	0.8087	0.3720	0.8435	0.7991	0.6733	0.2605
14	0.7998	0.7990	0.3518	0.8354	0.7891	0.6585	0.2414
15	0.7905	0.7897	0.3331	0.8275	0.7793	0.6442	0.2241
16	0.7815	0.7806	0.3156	0.8199	0.7699	0.6305	0.2082
17	0.7727	0.7718	0.2993	0.8125	0.7607	0.6172	0.1938
18	0.7641	0.7632	0.2841	0.8052	0.7517	0.6045	0.1805
19	0.7558	0.7548	0.2698	0.7981	0.7430	0.5922	0.1683
20	0.7476	0.7466	0.2565	0.7911	0.7345	0.5803	0.1570

21	0.7397	0.7386	0.2440	0.7844	0.7262	0.5688	0.1467
22	0.7319	0.7308	0.2322	0.7777	0.7181	0.5576	0.1371
23	0.7243	0.7232	0.2211	0.7712	0.7102	0.5468	0.1283
24	0.7169	0.7157	0.2107	0.7648	0.7024	0.5363	0.1201
25	0.7096	0.7084	0.2008	0.7585	0.6948	0.5262	0.1126
26	0.7024	0.7012	0.1915	0.7523	0.6874	0.5163	0.1055
27	0.6955	0.6942	0.1828	0.7463	0.6801	0.5067	0.0990
28	0.6886	0.6873	0.1745	0.7404	0.6730	0.4974	0.0930
29	0.6819	0.6805	0.1666	0.7345	0.6660	0.4883	0.0873
30	0.6753	0.6739	0.1592	0.7288	0.6592	0.4795	0.0821
31	0.6688	0.6674	0.1521	0.7231	0.6524	0.4709	0.0772
32	0.6624	0.6610	0.1455	0.7176	0.6458	0.4625	0.0726
33	0.6562	0.6547	0.1391	0.7121	0.6394	0.4543	0.0683
34	0.6500	0.6485	0.1331	0.7067	0.6330	0.4464	0.0644
35	0.6440	0.6425	0.1274	0.7014	0.6267	0.4386	0.0606
36	0.6380	0.6365	0.1220	0.6961	0.6206	0.4311	0.0571
37	0.6322	0.6306	0.1169	0.6910	0.6145	0.4237	0.0539
38	0.6264	0.6248	0.1120	0.6859	0.6086	0.4165	0.0508
39	0.6208	0.6192	0.1073	0.6809	0.6027	0.4095	0.0480
40	0.6152	0.6136	0.1028	0.6760	0.5970	0.4026	0.0453
41	0.6097	0.6080	0.0986	0.6711	0.5913	0.3959	0.0428
42	0.6043	0.6026	0.0946	0.6663	0.5858	0.3893	0.0404
43	0.5990	0.5973	0.0907	0.6615	0.5803	0.3829	0.0382
44	0.5938	0.5920	0.0871	0.6569	0.5749	0.3767	0.0361
45	0.5886	0.5868	0.0836	0.6522	0.5695	0.3705	0.0341
46	0.5835	0.5817	0.0802	0.6477	0.5643	0.3645	0.0323
47	0.5785	0.5766	0.0770	0.6432	0.5591	0.3587	0.0306
48	0.5735	0.5717	0.0740	0.6387	0.5540	0.3529	0.0289
49	0.5686	0.5668	0.0711	0.6343	0.5490	0.3473	0.0274
50	0.5638	0.5619	0.0683	0.6300	0.5441	0.3418	0.0260
51	0.5591	0.5572	0.0656	0.6257	0.5392	0.3364	0.0246
52	0.5544	0.5525	0.0631	0.6214	0.5344	0.3311	0.0233
53	0.5498	0.5478	0.0607	0.6173	0.5297	0.3260	0.0221
54	0.5452	0.5432	0.0583	0.6131	0.5250	0.3209	0.0209
55	0.5407	0.5387	0.0561	0.6090	0.5204	0.3160	0.0199
56	0.5363	0.5342	0.0540	0.6050	0.5158	0.3111	0.0188
57	0.5319	0.5298	0.0519	0.6010	0.5113	0.3063	0.0179
58	0.5275	0.5255	0.0500	0.5970	0.5069	0.3017	0.0170
59	0.5233	0.5212	0.0481	0.5931	0.5025	0.2971	0.0161
60	0.5190	0.5170	0.0463	0.5892	0.4982	0.2926	0.0153
61	0.5149	0.5128	0.0446	0.5854	0.4939	0.2882	0.0145
62	0.5107	0.5086	0.0429	0.5816	0.4897	0.2839	0.0138
63	0.5067	0.5045	0.0414	0.5778	0.4856	0.2797	0.0131
64	0.5027	0.5005	0.0398	0.5741	0.4815	0.2755	0.0125
65	0.4987	0.4965	0.0384	0.5705	0.4774	0.2714	0.0118

© 2025 Schaffer EM et al. *JAMA Network Open*

66	0.4948	0.4926	0.0370	0.5668	0.4734	0.2674	0.0113
67	0.4909	0.4887	0.0356	0.5632	0.4695	0.2635	0.0107
68	0.4870	0.4848	0.0344	0.5597	0.4656	0.2596	0.0102
69	0.4832	0.4810	0.0331	0.5561	0.4617	0.2559	0.0097
70	0.4795	0.4773	0.0319	0.5527	0.4579	0.2521	0.0092
71	0.4758	0.4735	0.0308	0.5492	0.4541	0.2485	0.0088
72	0.4721	0.4699	0.0297	0.5458	0.4504	0.2449	0.0084
73	0.4685	0.4662	0.0286	0.5424	0.4467	0.2414	0.0080
74	0.4649	0.4626	0.0276	0.5390	0.4431	0.2379	0.0076
75	0.4614	0.4591	0.0266	0.5357	0.4395	0.2346	0.0072
76	0.4579	0.4556	0.0257	0.5324	0.4359	0.2312	0.0069
77	0.4544	0.4521	0.0248	0.5292	0.4324	0.2279	0.0065
78	0.4510	0.4486	0.0239	0.5259	0.4290	0.2247	0.0062
79	0.4476	0.4452	0.0231	0.5227	0.4255	0.2216	0.0059
80	0.4443	0.4419	0.0223	0.5196	0.4221	0.2185	0.0057
81	0.4409	0.4386	0.0215	0.5164	0.4188	0.2154	0.0054
82	0.4377	0.4353	0.0208	0.5133	0.4154	0.2124	0.0051
83	0.4344	0.4320	0.0201	0.5102	0.4122	0.2095	0.0049
84	0.4312	0.4288	0.0194	0.5072	0.4089	0.2065	0.0047
85	0.4280	0.4256	0.0187	0.5042	0.4057	0.2037	0.0045
86	0.4249	0.4224	0.0181	0.5012	0.4025	0.2009	0.0043
87	0.4218	0.4193	0.0175	0.4982	0.3994	0.1981	0.0041
88	0.4187	0.4162	0.0169	0.4952	0.3963	0.1954	0.0039
89	0.4156	0.4132	0.0163	0.4923	0.3932	0.1928	0.0037
90	0.4126	0.4101	0.0158	0.4894	0.3902	0.1901	0.0035
91	0.4096	0.4071	0.0152	0.4866	0.3872	0.1876	0.0034
92	0.4067	0.4042	0.0147	0.4837	0.3842	0.1850	0.0032
93	0.4037	0.4012	0.0142	0.4809	0.3812	0.1825	0.0031
94	0.4008	0.3983	0.0138	0.4781	0.3783	0.1801	0.0029
95	0.3980	0.3954	0.0133	0.4753	0.3754	0.1776	0.0028
96	0.3951	0.3926	0.0129	0.4726	0.3726	0.1753	0.0027
97	0.3923	0.3898	0.0124	0.4698	0.3697	0.1729	0.0026
98	0.3895	0.3870	0.0120	0.4671	0.3669	0.1706	0.0024
99	0.3868	0.3842	0.0116	0.4645	0.3642	0.1684	0.0023
100	0.3840	0.3815	0.0112	0.4618	0.3614	0.1661	0.0022
101	0.3813	0.3788	0.0109	0.4592	0.3587	0.1639	0.0021
102	0.3787	0.3761	0.0105	0.4566	0.3560	0.1618	0.0020
103	0.3760	0.3734	0.0102	0.4540	0.3534	0.1597	0.0019
104	0.3734	0.3708	0.0099	0.4514	0.3507	0.1576	0.0019
105	0.3708	0.3682	0.0095	0.4488	0.3481	0.1555	0.0018
106	0.3682	0.3656	0.0092	0.4463	0.3455	0.1535	0.0017
107	0.3656	0.3630	0.0089	0.4438	0.3430	0.1515	0.0016
108	0.3631	0.3605	0.0086	0.4413	0.3405	0.1495	0.0016
109	0.3606	0.3580	0.0084	0.4388	0.3379	0.1476	0.0015
110	0.3581	0.3555	0.0081	0.4364	0.3355	0.1457	0.0014

© 2025 Schaffer EM et al. *JAMA Network Open*

111	0.3556	0.3530	0.0078	0.4340	0.3330	0.1438	0.0014
112	0.3532	0.3506	0.0076	0.4315	0.3306	0.1419	0.0013
113	0.3508	0.3481	0.0073	0.4292	0.3282	0.1401	0.0012
114	0.3484	0.3457	0.0071	0.4268	0.3258	0.1383	0.0012
115	0.3460	0.3434	0.0069	0.4244	0.3234	0.1366	0.0011
116	0.3437	0.3410	0.0067	0.4221	0.3211	0.1348	0.0011
117	0.3413	0.3387	0.0065	0.4198	0.3187	0.1331	0.0010
118	0.3390	0.3363	0.0063	0.4175	0.3164	0.1314	0.0010
119	0.3367	0.3341	0.0061	0.4152	0.3142	0.1297	0.0010
120	0.3345	0.3318	0.0059	0.4129	0.3119	0.1281	0.0009
121	0.3322	0.3295	0.0057	0.4107	0.3097	0.1265	0.0009
122	0.3300	0.3273	0.0055	0.4084	0.3075	0.1249	0.0008
123	0.3278	0.3251	0.0053	0.4062	0.3053	0.1233	0.0008
124	0.3256	0.3229	0.0052	0.4040	0.3031	0.1218	0.0008
125	0.3234	0.3207	0.0050	0.4018	0.3009	0.1203	0.0007
126	0.3213	0.3186	0.0049	0.3997	0.2988	0.1188	0.0007
127	0.3191	0.3164	0.0047	0.3975	0.2967	0.1173	0.0007
128	0.3170	0.3143	0.0046	0.3954	0.2946	0.1158	0.0007
129	0.3149	0.3122	0.0044	0.3933	0.2925	0.1144	0.0006
130	0.3128	0.3101	0.0043	0.3912	0.2904	0.1130	0.0006
131	0.3108	0.3081	0.0042	0.3891	0.2884	0.1116	0.0006
132	0.3087	0.3060	0.0040	0.3870	0.2864	0.1102	0.0006
133	0.3067	0.3040	0.0039	0.3850	0.2844	0.1088	0.0005
134	0.3047	0.3020	0.0038	0.3829	0.2824	0.1075	0.0005
135	0.3027	0.3000	0.0037	0.3809	0.2804	0.1062	0.0005
136	0.3007	0.2980	0.0036	0.3789	0.2785	0.1049	0.0005
137	0.2988	0.2960	0.0035	0.3769	0.2765	0.1036	0.0004
138	0.2968	0.2941	0.0034	0.3749	0.2746	0.1023	0.0004
139	0.2949	0.2922	0.0032	0.3729	0.2727	0.1011	0.0004
140	0.2930	0.2903	0.0032	0.3710	0.2708	0.0999	0.0004
141	0.2911	0.2884	0.0031	0.3690	0.2690	0.0987	0.0004
142	0.2892	0.2865	0.0030	0.3671	0.2671	0.0975	0.0004
143	0.2874	0.2846	0.0029	0.3652	0.2653	0.0963	0.0003
144	0.2855	0.2828	0.0028	0.3633	0.2635	0.0951	0.0003
145	0.2837	0.2809	0.0027	0.3614	0.2617	0.0940	0.0003
146	0.2819	0.2791	0.0026	0.3595	0.2599	0.0929	0.0003
147	0.2801	0.2773	0.0025	0.3576	0.2581	0.0917	0.0003
148	0.2783	0.2755	0.0025	0.3558	0.2564	0.0906	0.0003
149	0.2765	0.2738	0.0024	0.3540	0.2546	0.0896	0.0003
150	0.2748	0.2720	0.0023	0.3521	0.2529	0.0885	0.0003
151	0.2730	0.2703	0.0023	0.3503	0.2512	0.0874	0.0003
152	0.2713	0.2685	0.0022	0.3485	0.2495	0.0864	0.0002
153	0.2696	0.2668	0.0021	0.3467	0.2478	0.0854	0.0002
154	0.2679	0.2651	0.0021	0.3450	0.2461	0.0844	0.0002
155	0.2662	0.2634	0.0020	0.3432	0.2445	0.0834	0.0002

156	0.2645	0.2618	0.0019	0.3414	0.2428	0.0824	0.0002
157	0.2628	0.2601	0.0019	0.3397	0.2412	0.0814	0.0002
158	0.2612	0.2584	0.0018	0.3380	0.2396	0.0804	0.0002
159	0.2596	0.2568	0.0018	0.3363	0.2380	0.0795	0.0002
160	0.2579	0.2552	0.0017	0.3346	0.2364	0.0786	0.0002
161	0.2563	0.2536	0.0017	0.3329	0.2348	0.0776	0.0002
162	0.2547	0.2520	0.0016	0.3312	0.2333	0.0767	0.0002
163	0.2531	0.2504	0.0016	0.3295	0.2317	0.0758	0.0002
164	0.2516	0.2488	0.0015	0.3279	0.2302	0.0750	0.0001
165	0.2500	0.2473	0.0015	0.3262	0.2286	0.0741	0.0001
166	0.2485	0.2457	0.0015	0.3246	0.2271	0.0732	0.0001
167	0.2469	0.2442	0.0014	0.3229	0.2256	0.0724	0.0001
168	0.2454	0.2427	0.0014	0.3213	0.2242	0.0715	0.0001
169	0.2439	0.2411	0.0013	0.3197	0.2227	0.0707	0.0001
170	0.2424	0.2396	0.0013	0.3181	0.2212	0.0699	0.0001
171	0.2409	0.2382	0.0013	0.3165	0.2198	0.0691	0.0001
172	0.2394	0.2367	0.0012	0.3150	0.2183	0.0683	0.0001
173	0.2379	0.2352	0.0012	0.3134	0.2169	0.0675	0.0001
174	0.2365	0.2338	0.0012	0.3118	0.2155	0.0667	0.0001
175	0.2350	0.2323	0.0011	0.3103	0.2141	0.0660	0.0001
176	0.2336	0.2309	0.0011	0.3088	0.2127	0.0652	0.0001
177	0.2322	0.2295	0.0011	0.3072	0.2113	0.0645	0.0001
178	0.2308	0.2280	0.0010	0.3057	0.2099	0.0637	0.0001
179	0.2294	0.2266	0.0010	0.3042	0.2086	0.0630	0.0001
180	0.2280	0.2253	0.0010	0.3027	0.2072	0.0623	0.0001

© 2025 Schaffer EM et al. *JAMA Network Open*

eReferences

1. Stevenson LW, Miller LW, Desvigne-Nickens P, et al. Left ventricular assist device as destination for patients undergoing intravenous inotropic therapy: a subset analysis from REMATCH (Randomized Evaluation of Mechanical Assistance in Treatment of Chronic Heart Failure). *Circulation*. Aug 24 2004;110(8):975-81. doi:10.1161/01.CIR.0000139862.48167.23
2. Starling RC, Estep JD, Horstmanshof DA, et al. Risk Assessment and Comparative Effectiveness of Left Ventricular Assist Device and Medical Management in Ambulatory Heart Failure Patients: The ROADMAP Study 2-Year Results. *JACC Heart Fail*. Jul 2017;5(7):518-527. doi:10.1016/j.jchf.2017.02.016
3. Mehra MR, Goldstein DJ, Cleveland JC, et al. Five-Year Outcomes in Patients With Fully Magnetically Levitated vs Axial-Flow Left Ventricular Assist Devices in the MOMENTUM 3 Randomized Trial. *JAMA*. Sep 27 2022;328(12):1233-1242. doi:10.1001/jama.2022.16197
4. Malhotra C, Foo R, Singh R, et al. Study protocol for a cohort study of patients with advanced heart failure in Singapore. *BMJ Open*. Sep 17 2018;8(9):e022248. doi:10.1136/bmjopen-2018-022248
5. Samson D. *Special report: cost-effectiveness of left-ventricular assist devices as destination therapy for end-stage heart failure.* Vol. 19. 2004:1. *Technol Eval Cent Assess Program Exec Summ*.
6. Rogers JG, Bostic RR, Tong KB, Adamson R, Russo M, Slaughter MS. Cost-effectiveness analysis of continuous-flow left ventricular assist devices as destination therapy. *Circ Heart Fail*. Jan 2012;5(1):10-6. doi:10.1161/circheartfailure.111.962951
7. Neyt M, Van den Bruel A, Smit Y, et al. Cost-effectiveness of continuous-flow left ventricular assist devices. *Int J Technol Assess Health Care*. Jul 2013;29(3):254-60. doi:10.1017/s0266462313000238
8. Long EF, Swain GW, Mangi AA. Comparative survival and cost-effectiveness of advanced therapies for end-stage heart failure. *Circ Heart Fail*. May 2014;7(3):470-8. doi:10.1161/circheartfailure.113.000807
9. Chew DS, Manns B, Miller RJH, Sharma N, Exner DV. Economic Evaluation of Left Ventricular Assist Devices for Patients With End Stage Heart Failure Who Are Ineligible for Cardiac Transplantation. *Can J Cardiol*. Oct 2017;33(10):1283-1291. doi:10.1016/j.cjca.2017.07.012
10. Silvestry SC, Mahr C, Slaughter MS, et al. Cost-Effectiveness of a Small Intrapericardial Centrifugal Left Ventricular Assist Device. *ASAIO J*. Aug 2020;66(8):862-870. doi:10.1097/MAT.0000000000001211
11. Schueler S, Silvestry SC, Cotts WG, et al. Cost-effectiveness of left ventricular assist devices as destination therapy in the United Kingdom. *ESC Heart Fail*. Aug 2021;8(4):3049-3057. doi:10.1002/ehf2.13401
12. Lim HS, Shaw S, Carter AW, Jayawardana S, Mossialos E, Mehra MR. A clinical and cost-effectiveness analysis of the HeartMate 3 left ventricular assist device for transplant-ineligible patients: A United Kingdom perspective. *J Heart Lung Transplant*. Feb 2022;41(2):174-186. doi:10.1016/j.healun.2021.11.014
