# Supplementary material for: Cost-Effectiveness of Left Ventricular Assist Device for Transplant-Ineligible Patients
Source: JAMA Netw Open. 2025 Apr 18;8(4):e254483. doi: 10.1001/jamanetworkopen.2025.4483 (PMC12008763; doi:10.1001/jamanetworkopen.2025.4483)
Supplement: Supplement 2. — Data Sharing Statement [file jamanetwopen-e254483-s002.pdf]

Data Sharing Statement

Schaffer. Cost-Effectiveness of Left Ventricular Assist Device for Transplant-Ineligible Patients. *JAMA Netw Open*. Published April 18, 2025. doi:10.1001/jamanetworkopen.2025.4483

Data
Data available: Yes
Data types: Deidentified participant data, Data (not involving human participants), Data dictionary
How to access data: Data will be made available upon reasonable request by contacting the corresponding author (elisabeth.schaffer@nus.edu.sg).
When available: With publication

Supporting Documents
Document types: None

Additional Information
Who can access the data: Anyone requesting the data
Types of analyses: For any purpose
Mechanisms of data availability: Depends on study purpose
